# The Effect of Ondansetron on the Analgesic Efficacy of Tramadol in Patients Undergoing Laparoscopic Cholecystectomy

**DOI:** 10.1155/2022/7387600

**Published:** 2022-03-08

**Authors:** Sevgi Bilgen, Dilek Erdoğan Ari, Emel Özveri

**Affiliations:** ^1^Acibadem Kozyataği Hospital, Department of Anesthesiology, Ondokuz Mayıs Mah, Begonya Sokak, No. 12, Kadıköy, İstanbul, Turkey; ^2^Acibadem Kozyataği Hospital, Department of General Surgery, Ondokuz Mayıs Mah, Begonya Sokak, No. 12, Kadıköy, İstanbul, Turkey

## Abstract

**Purpose:**

Investigating the effect of ondansetron on the efficacy of tramadol in patients undergoing laparoscopic cholecystectomy.

**Methods:**

Sixty American Society of Anesthesiologists (ASA) I-II patients over the age of 18 who underwent laparoscopic cholecystectomy were included in this study. All patients were given 1 mg/kg tramadol intravenously (iv) during the intraoperative period. Patients were randomly assigned to receive either 4 mg ondansetron (Group O) or 2 mL saline (Group S). Postoperative tramadol consumption, pain score (NRS), intensity of nausea (NRS), presence of vomiting, consumption of rescue analgesics and antiemetics, and patient satisfaction were recorded.

**Results:**

A total of 60 patients were enrolled in the study; five patients were excluded due to deviation from the protocol. Data from 55 patients (Group O: 28 patients, Group S: 27 patients) were evaluated in the study. No differences between the two groups were detected for postoperative consumption of tramadol, pain score (NRS), intensity of nausea (NRS), presence of vomiting, consumption of rescue analgesics and antiemetics, and patient satisfaction.

**Conclusions:**

The results showed that coadministration of tramadol and ondansetron did not change tramadol consumption during the postoperative 24 hours after laparoscopic cholecystectomy. Clinical trial registration number is as follows: https://clinicaltrials.gov/ct2/show/NCT04745273—01/31/2021.

## 1. Introduction

Pain, nausea, and vomiting are unpleasant consequences that reduce patient comfort and may lead to various complications in the postoperative period. Various drugs combinations are used to prevent these undesirable results. However, the combination of drugs may not always be completely beneficial [1].

The popularity of tramadol use is increasing due to the fact that side effects such as respiratory depression, opioid addiction, and abuse are less expected from those with tramadol than other opioids [2, 3]. Tramadol also has the advantage of having a lower risk of hemodynamic instability and gastrointestinal inhibition [3]. As a result, tramadol is preferred for postoperative analgesia following abdominal surgery [3]. On the other hand, nausea and vomiting are common side effects of tramadol [4, 5].

Serotonin (5-hydroxytryptamine [5-HT]) subtype 3 (5-HT_3_) receptor antagonists, which inhibit the stimulatory effect of serotonin on the afferent vagal nerve pathway and on the chemoreceptor trigger zone, are among the drugs of choice in patients at moderate to high risk for postoperative nausea and vomiting (PONV) [6]. The efficacy, safety, and cost-effectiveness of these drugs for PONV prophylaxis when used as monotherapy at the end of anesthesia have been demonstrated [7]. Ondansetron, which we have preferred to administer to examine its impact on the effect of tramadol, is also a 5-HT_3_ receptor antagonist that is frequently used for PONV prevention [8].

Various studies proclaim that there is a drug interaction between these two drugs and that ondansetron reduces the effects of tramadol, causing increased analgesic requirements in the postoperative period [9–11]. In some of these studies, the combination of tramadol and ondansetron is not recommended [9, 12]. Other studies, in contrast, showed no interaction between these two drugs [13, 14].

The aim of this study was to evaluate whether there is an impact of ondansetron on the analgesic effect of tramadol in patients undergoing cholecystectomy or not. The hypothesis of the study was that if ondansetron was given together with tramadol perioperatively, tramadol consumption would increase postoperatively.

## 2. Materials and Methods

The study was registered with the https://www.clinicaltrials.gov protocol registration system (https://clinicaltrials.gov/ct2/show/NCT04745273—01/31/2021) and was approved by the Clinical Research Ethics Committee of Acıbadem University, Istanbul, Turkey (No: 2020–12/03). Written informed consent was obtained from all study participants. Sixty American Society of Anesthesiologist's Class I or II patients, over age 18, undergoing laparoscopic cholecystectomy under general anesthesia were included in this single center, double-blind, randomized, controlled trial. Patients were excluded if they had contraindication for the studied medications, were pregnant or breastfeeding, had inability of using or understanding the patient-controlled analgesia (PCA) device, received an opioid or antiemetic drug within 24 hours before surgery, and had a history of mental illness, epilepsy, alcohol, or substance abuse.

The patients were informed on how to use the PCA device and the 0-to-10-point numerical rating scale (NRS) to assess pain (0: no pain, 10: worst pain imaginable) and nausea intensity (0: no nausea, 10: nausea as bad as it could be) before the surgery.

All patients were premedicated with intravenous (iv) midazolam (2 mg) about 15 minutes before the induction of anesthesia. Heart rate, noninvasive blood pressure, and oxygen saturation on pulse oximetry were monitored on arrival to the operating room. Anesthesia was induced with iv propofol (2 mg/kg) and fentanyl (2 *μ*g/kg). Muscle relaxation was provided by 0.6 mg/kg rocuronium. After tracheal intubation, general anesthesia was maintained with sevoflurane (1 minimum alveolar concentration) in an oxygen/air mixture and remifentanil infusion (0.1–0.3 *μ*g/kg/min). Normocapnic mechanical ventilation was performed after intubation. The nasogastric tube was placed in all patients and removed at the end of the surgery. All surgeries were performed by the same surgical team. Pneumoperitoneum was created by insufflation of CO_2_ and the intraabdominal pressure was set to maximum 12 mmHg. At the end of the surgery, 0.5% bupivacaine (20 ml) was applied to the incision sites of all patients by the surgical team.

All patients received tramadol (1 mg/kg), paracetamol (1 g), and dexketoprofen (50 mg) intravenously about 30 minutes before emergence. Patients were randomized into two study groups (30 patients in each group), using a computer-generated random number table by an anesthesiologist who did not participate in the study. Ondansetron 4 mg iv (2 mL) was given to the ondansetron group (Group O), and 0.9% saline 2 mL iv was given to the saline group (Group S) after the administration of tramadol. Study drugs were prepared by an anesthesiologist independent of the study. The patients, care givers, and those recording the outcomes were blinded to group assignment. After the surgery, muscle relaxation was reversed by administering sugammadex (2–4 mg/kg). After return of spontaneous ventilation and tracheal extubation, patients were transferred to the recovery room.

Apfel's risk factor, duration of surgery, and anesthesia were recorded. The patient's heart rate, blood pressure, oxygen saturation, sedation score, level of pain (with NRS), degree of nausea (with NRS), whether there is vomiting, whether rescue analgesic or antiemetic treatment was required, and the amount of tramadol use during the postoperative 24 hours were recorded at 1, 2, 4, 8, 12, and 24 hours in the postoperative period. Sedation status was evaluated with the Ramsay score (Table 1). Complete response to antiemetic prophylaxis or therapy was defined as NRS ≤4 for nausea, no vomiting, and no need for rescue antiemetics.

Postoperative analgesia was provided with the PCA technique by using iv tramadol (4 mg/mL, total dose: 400 mg) (2.5 mL bolus and 10 minutes lockout interval without basal infusion). At arrival to the recovery room, all patients were connected to the PCA device. An anesthesiologist asked each patient about their level of pain and nausea. If the NRS pain score >4, tramadol 20 mg iv was administered. The assessment was repeated after 5 minutes. If the pain level was still >4, 20 mg tramadol was added. After 5 minutes, if the patient's pain persisted, 50 mcg iv fentanyl was administered. Patients in both groups were given iv 1 g paracetamol every 8 hours in the postoperative period. After discharge from the recovery room, diclofenac sodium 75 mg intramuscular (im) was given as a rescue analgesic to the patients if NRS for pain is >4. Patients were treated with iv 10 mg metoclopramide postoperatively if the patient had nausea >4, vomited, or if the patient requested it.

Patient's satisfaction with pain management was questioned 24 hours after surgery: 1: I am not satisfied 2: I am partially satisfied 3: I am generally satisfied 4: I am completely satisfied.

### 2.1. Statistical Analysis

The primary outcome of the study was total tramadol consumption. With a power of 80% and an alpha error of 5%, for *d* = 0.8, the sample size calculation determined that 26 patients were required for each group, using the G ∗ Power (v3.1.7) program. Considering the possibility of drop out and lack of data, the total number of patients required for the study was determined to be 60.

We analyzed the data with SPSS version 16 (SPSS Inc., Chicago, Illinois, USA). Convenience of parameters to the normal distribution was assessed with Shapiro Wilks test while assessing the study data. Student *t*-test was used in comparing quantitative data for comparing parameters that showed normal distribution between the two groups, while Mann-Whitney *U* test was used in comparing data that did not show a normal distribution between the two groups. Chi-square test and Fisher's exact test were used in comparing qualitative parameters. *p* ≤ 0.005 was considered statistically significant.

## 3. Results

A total of 60 patients were enrolled in the study; five patients were excluded due to deviation from the protocol, leaving 55 patients for evaluation. Of these, 28 were in group O, and 27 were in group S.

The age, weight, height, male/female ratio, and the distribution of ASA and Apfel's risk score were similar in both groups (Table 2). There were no differences in sedation scores at any interval during the postoperative period.

**Figure 1:**
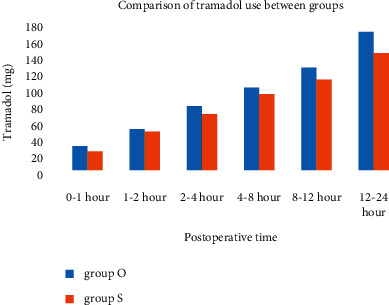
Consumption of tramadol (in milligrams) during the postoperative 24 hours showed no difference between groups. Group O, *n* = 28; Group S, *n* = 27.

Consumption of tramadol during the postoperative period showed no difference between groups (Figure 1). Median total tramadol consumption was 150 (Interquartile range (IQR): 420) mg in the ondansetron group and 120 (380) mg in the saline group (*p*=0.407).

One patient required fentanyl 50 mcg iv in both of groups in the recovery room. After discharge from the recovery room, diclofenac sodium 75 mg im was given to one patient from each group as a rescue analgesic. The patients who received diclofenac sodium were different from the patients who needed fentanyl.

Duration of surgery (44.00 ± 17.00 vs. 32.29 ± 14.68 min) and duration of anesthesia (63.14 ± 16.62 vs. 51.25 ± 15.16 min) were shorter in Group S than Group O (*p*=0.002 and *p*=0.001 respectively) (Table 2).

**Figure 2:**
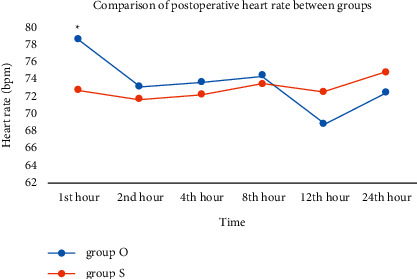
The heart rate did not differ significantly between the groups, except for the first postoperative hour: 78.64/min in group O versus 72.74/min in Group S (*p*=0.020). Group O, *n* = 28; Group S, *n* = 27.

The heart rate did not differ significantly between the groups, except for the first postoperative hour: 78.64/min in group O versus 72.74/min in Group S (*p*=0.020) (Figure 2). The systolic and diastolic arterial pressure showed no difference between groups during the postoperative period.

**Figure 3:**
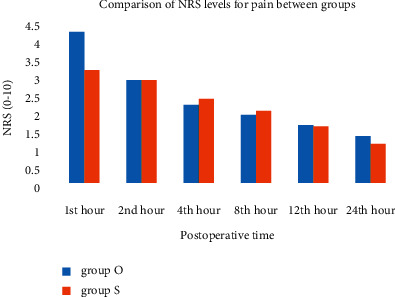
Pain score on numerical rating scale (NRS) (0–10) in the postoperative 24 hours. There was no difference between the two groups during the study period. Group O, *n* = 28; Group S, *n* = 27.

**Figure 4:**
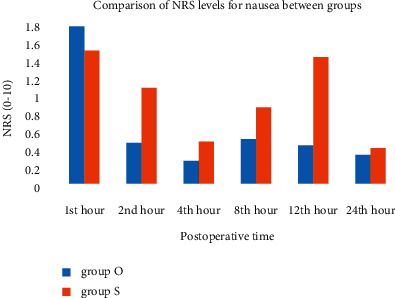
Nausea score on numerical rating scale (NRS) (0–10) in the postoperative 24 hours. There was no difference between the two groups during the study period. Group O, *n* = 28; Group S *n* = 27.

The incidence of postoperative nausea and vomiting showed no difference between groups (Table 3). The requirements of rescue antiemetic and complete response were similar between groups (Table 3). The NRS for pain (Figure 3) (Table 4) and the NRS for nausea (Figure 4) (Table 4) were similar in both groups.

Satisfaction of the patients showed no difference between groups (Table 2).

## 4. Discussion

Coadministration of tramadol and ondansetron did not change tramadol consumption during the postoperative 24 hours after laparoscopic cholecystectomy.

It is possible to obtain more successful results with multimodal analgesia in postoperative pain management. Tramadol, a centrally acting analgesic, has an analgesic effect with both opioid and nonopioid action mechanisms [15]. Its multimodal mechanism of action and the presence of its active metabolite make tramadol one of the preferred analgesics to prevent postoperative pain as a part of multimodal analgesia. Ondansetron is a serotonin (5-HT3) receptor antagonist, and it can be used effectively in PONV. But it is assumed that there is an interaction between the two drugs due to two mechanisms: pharmacokinetic and pharmacodynamic interaction [1, 5]. However, the results of clinical studies on the coadministration of tramadol and ondansetron are controversial [9, 10, 13, 14]. In some of these studies, the authors found that there is an interaction between tramadol and ondansetron; therefore, tramadol consumption increases due to the coadministration of ondansetron [9–11, 16]. These studies include very different patient groups such as neck dissection or mastoidectomy [9], lumbar laminectomy [10], lower extremity bone surgery [11], nonlaparoscopic hernioplasty, or thyroidectomy [16]. The results of these studies are consistent with our hypothesis. However, unlike these studies, we conducted our study in a completely different group of patients who were scheduled for laparoscopic cholecystectomy. Other studies found that ondansetron has no effect on tramadol consumption [13, 14]. Stevens et al. published a systematic review and meta-analysis that evaluated a drug interaction between tramadol and ondansetron [1]. The authors concluded that there is an interaction between tramadol and ondansetron, and this interaction results in a reduction in the efficacy of tramadol [1]. However, the results of a study included in this meta-analysis, which is one of the most comprehensive studies on the subject involving 179 patients, contradict the findings of other studies [13]. Rauers et al. administered tramadol 3 mg/kg (maximum 250 mg) and dipyrone 1 g iv intraoperatively in their study on patients scheduled for elective abdominal surgery [13]. It is possible to think that this high dose of tramadol overcomes the antagonistic effect of ondansetron. We preferred to use 1 mg/kg tramadol as a loading dose together with intraoperative nonopioid analgesics in our study. Because we thought that the use of nonopioid analgesics could increase the success of postoperative analgesia by reducing tramadol consumption and opioid-related side effects such as PONV. Despite the low dose of tramadol, our results were correlated with this study, which evaluated the interaction of these two drugs and showed that coadministration of ondansetron with tramadol did not reduce the analgesic effect of tramadol.

Different results have been obtained not only in clinical studies, but also in animal studies. Dursteler et al., in their study on mice, showed an antagonistic interaction on antinociception between tramadol and ondansetron [12]. Relying on the results of their study, Dursteler et al. suggested to avoid using these drug combinations in humans for postoperative analgesia. However, they found that ondansetron, when used alone, was seven times more potent than tramadol on antinociception depending on the type of stimulus. They observed that ondansetron caused an increase in pain tolerance in the presence of chemical stimuli in female mice [12]. The results of another animal study conducted by Erhan et al. are completely different. Erhan et al. showed that ondansetron did not decrease the analgesic effectiveness of tramadol in mice [17]. Besides, they found that different doses of ondansetron alone had no effect on the pain threshold levels of the mice [17].

Tramadol consists of two different enantiomers. These are (+)-tramadol and (−)-tramadol [3]. The (+)-enantiomer inhibits serotonin (5-hydroxytryptamine, 5-HT) reuptake and activates its release, while (−)-tramadol inhibits norepinephrine reuptake and activates adrenergic receptors [3]. Because tramadol increases 5-HT level in the central nervous system, spinal cord, brain, and probably in the periphery, the serotonergic system has been suggested to be involved in tramadol analgesia [12, 18].

Ondansetron, a selective serotonin receptor antagonist, inhibits the stimulatory effect of serotonin on the afferent vagal nerve pathway. In studies suggesting that there is an interaction between ondansetron and tramadol, it is argued that the decrease in the analgesic effect of tramadol is due to the competitive antagonism of ondansetron on the 5-HT3 receptors at the spinal level [9, 16]. In our study, there was no increase in tramadol consumption due to the use of ondansetron. In this case, it should be remembered that the analgesic effect of tramadol is not only on the serotonergic system, but also on opioid receptors.

O-Desmethyltramadol (M1), the effective metabolite of tramadol, affects opioid receptors [19]. There are various studies supporting that the M1 metabolite creates the opioid effect of tramadol and thus has a significant effect on its analgesic effect [16, 19, 20]. The O-demethylation of tramadol to M1 is catalyzed by cytochrome P450 (CYP) 2D6 [3]. Therefore, the metabolization of tramadol to its active metabolite M1 depends on the CYP2D6 enzyme. There are significant differences between individuals in the activity and amount of the CYP2D6 enzyme due to genetic polymorphisms [5, 21]. The individuals may be divided into four types regarding to the metabolization capacity of CYP2D6: 1- poor metabolizer (little or no CYP2D6 function), 2- intermediate metabolizer (metabolized drugs at a rate between poor and extensive metabolizers), 3- extensive metabolizer (normal CYP2D6 function), and 4- ultra-rapid metabolizer (greater than normal CYP2D6 function) [5]. Therefore, tramadol is metabolized to its active metabolite M1 rapidly in some individuals (ultra-rapid metabolizers) and slowly in others (poor metabolizers). In this case, it is thought that the differences in CYP2D6 polymorphism in patients may affect the analgesic efficacy, amount of consumption, and side effects of tramadol. Not only tramadol, but also ondansetron is metabolized by CYP2D6 [5]. It is accepted that the interaction between the two drugs may be due to the competition for the CYP2D6 enzyme [1, 22]. However, Rauers et al. have previously analyzed the CYP2D6 genetic variants of patients in their study on the antagonistic effect of ondansetron and tramadol. While they found a correlation between the active metabolite M1 and CYP2D6 genotypes in patients, they did not find the same effect with ondansetron [13]. They concluded that the analgesic effect of tramadol after major abdominal surgery is not reduced by the combination of tramadol with ondansetron [13]. Similar to the results of Rauers et al., we did not observe an increase in tramadol consumption due to the coadministration of ondansetron. Since CYP2D6 enzyme levels were not known because gene analysis was not performed in our study, it was not possible for us to know the role of its active metabolite on the analgesic effect of tramadol and also the antiemetic effect of ondansetron. The results of the study of Rauers et al. suggest that postoperative pain management can be reinforced by selecting agents that are compatible with the CYP2D6 genotype of patients [13]. In a study conducted on 92 Turkish breast cancer patients, CYP2D6 gene polymorphisms were classified as follows: 2.17% poor metabolizer, 11.95% intermediate metabolizer, 80.43% normal metabolizer, and 5.43% ultra-rapid metabolizer [23]. Nevertheless, it would not be acceptable to make a definitive judgment about the rates of gene polymorphism in the Turkish population based on the results of the study conducted on a limited and specific population. Unfortunately, in today's conditions, routine pharmacogenetic testing in patients seems unlikely, given the cost.

Concomitant use of nonopioid analgesics with tramadol may increase the success rate in postoperative analgesia [3, 5]. Thus, the incidence of opioid-induced side effect can be reduced [6]. We preferred to administer tramadol (1 mg/kg) together with paracetamol (1 g) and dexketoprofen (50 mg) in the perioperative period. In our study, additional analgesics other than tramadol were required in only two patients in each group.

In this study, a complete response for PONV was similar among the ondansetron and saline groups during the postoperative period. The causes of PONV are multifactorial. These factors may be female gender, nonsmoking status, history of PONV or motion sickness, type of surgery, longer duration of surgery, the use of inhalation anesthetic agents and nitrous oxide, reversal of neuromuscular blockade, postoperative pain, and use of postoperative opioid [24, 25]. In this study, the duration of surgery was longer in the ondansetron group than in the saline group. For this reason, better results may have been obtained in the ondansetron group in the prevention of PONV if the duration of surgery was similar to the saline group. Although some studies have shown that ondansetron was not successful in preventing PONV [10, 26], as a result of our study, it would not be appropriate to state that ondansetron is ineffective in preventing PONV. Treatment with a drug of different class is recommended for patients in whom prophylaxis with 5-HT3 receptor antagonists has failed within 6 hours of surgery [27]. Metoclopramide was preferred as a rescue antiemetic during the postoperative period in this study because it reduces the possibility of postoperative ileus and abdominal discomfort caused by opioids [6].

Although ondansetron is frequently used for PONV, it has various side effects. Cardiac side effects such as chest pain, electrocardiographic changes, hypotension, and tachycardia may occur, rarely associated with ondansetron [7, 28]. In our study, the heart rate of the patients was statistically higher in the ondansetron group at the first hour after surgery. There was no difference in the following hours. In our study, it can be thought that this may have been a side effect of ondansetron.

The main limitation of the present study is that pharmacogenetic tests were not performed on the patients. Another limitation of the study is that it was in a single center with a small number of patients. Despite these limitations, we believe that our study would pave the path for future systematic studies addressing the issue of interaction between tramadol and ondansetron.

## 5. Conclusion

In conclusion, we were unable to demonstrate that ondansetron had an effect on the analgesic efficacy of tramadol following laparoscopic cholecystectomy. However, to definitively determine whether ondansetron has an effect on tramadol or not, this topic needs further evaluation with extensive studies assessing the analysis of genetic polymorphism.

## Figures and Tables

**Table 1:** Ramsay score.

0	Patient is paralyzed, unable to assess the level of sedation
1	Patient is anxious, agitated, or restless
2	Patient is cooperative, oriented, and tranquil
3	Patient is sedated but responds to commands
4	Patient is asleep but responds to glabellar tap
5	Patient is asleep but responds to nail bed pressure
6	Patient is asleep, with no response to nail bed pressure

**Table 2:** Demographics characteristics, Apfel's risk score, duration of surgery and anesthesia, and patient satisfaction in both groups.

	Group O (*n* = 28), mean ± SD	Group S (*n* = 27), mean ± SD	*p* value
Age (year)	46.82 ± 11.30	47.48 ± 12.63	0.86 (1)
Weight (kg)	77.64 ± 19.49	78.25 ± 15.00	0.76 (1)
Height (cm)	166.64 ± 8.20	169.70 ± 10.64	0.11 (1)
Male/female	8/20	11/16	0.403 (2)
ASA I/ASA II	6/22	8/19	0.547 (2)
Apfel's risk score: 0/1/2/3/4	0/3/13/11/1	0/3/16/8/0	0.522 (3)
Duration of surgery (min)	44.00 ± 17.00	32.29 ± 14.68	0.002^*∗*^ (1)
Duration of anesthesia (min)	63.14 ± 16.62	51.25 ± 15.16	0.001^*∗*^ (1)
Patient satisfaction: 1/2/3/4	0/4/8/16	0/1/8/18	0.387 (3)

Group O: ondansetron group; Group S: saline group; ASA: American Society of Anesthesiologist. (1): Mann-Whitney *U* Test; (2): Fisher's exact test; (3): chi-square test ^*∗*^Statistically significant (*p* < 0.05).

**Table 3:** Incidence of postoperative nausea, vomiting, the use of rescue antiemetic, and complete response in both groups.

	Group O (*n* = 28)	Group S (*n* = 27)	*p* value
*Nausea (NRS* > 4)			
0–1 h	8 (28%)	6 (22%)	0.547 (2)
1–2 h	0 (0%)	3 (11%)	0.111 (2)
2–4 h	1 (3%)	1 (3%)	1.0 (2)
4–8 h	2 (7%)	3 (11%)	1.0 (2)
8–12 h	2 (7%)	4 (14%)	0.729 (2)
12–24 h	1 (3%)	1 (3%)	1.0 (2)
0–24 h	10 (35%)	12 (44%)	0.789 (2)

*Vomiting*			
0–1 h	0 (0%)	0 (0%)	—
1–2 h	0 (0%)	0 (0%)	—
2–4 h	0 (0%)	0 (0%)	—
4–8 h	1 (3%)	5 (18%)	0.101 (2)
8–12 h	0 (0%)	2 (7%)	0.111 (2)
12–24 h	0 (0%)	1 (3%)	0.491 (2)
0–24 h	1(3%)	7 (26%)	0.78 (2)

*Rescue antiemetic*			
0–1 h	8 (28%)	6 (22%)	0.758 (2)
1–2 h	0 (0%)	3 (11%)	0.111 (2)
2–4 h	1 (3%)	1 (3%)	1.0 (2)
4–8 h	3 (10%)	5 (18%)	0.469 (2)
8–12 h	2 (7%)	7 (26%)	0.078 (2)
12–24 h	2 (7%)	3 (11%)	0.669 (2)
0–24 h	10 (35%)	14 (51%)	0.423 (2)

*Complete response*			
0–1 h	20 (71%)	21(77%)	0.758 (2)
1–2 h	28 (100%)	24 (88%)	0.111 (2)
2–4 h	27 (96%)	26 (96%)	1.0 (2)
4–8 h	25 (89%)	22 (81%)	0.469 (2)
8–12	26 (92%)	20 (74%)	0.078 (2)
12–24 h	26 (92%)	24 (88%)	0.669 (2)
0–24 h	18 (64%)	13 (48%)	0.282 (2)

Group O: ondansetron group; Group S: saline group; NRS: numeric rating scale (2): Fisher's exact test.

**Table 4:** Comparison on NRS pain and nausea score during postoperative 24 h in both groups.

	Group O (*n* = 28), mean ± SD	Group S (*n* = 27), mean ± SD	Mean difference (95% CI) (group O–S)
*NRS pain score*			
0-1 h	4.21 ± 2.71	3.14 ± 2.90	1.06 (−0.45, 2.58)
1–2 h	2.85 ± 2.06	2.85 ± 2.01	0.00 (−1.09, 1.10)
2–4 h	2.17 ± 1.88	2.33 ± 1.92	−0.15 (−1.18, 0.87)
4–8 h	1.89 ± 1.89	2.00 ± 1.96	−0.10 (−1.14, 0.93)
8–12 h	1.60 ± 1.49	1.48 ± 1.31	0.12 (−0.63, 0.88)
12–24 h	1.32 ± 1.86	1.07 ± 1.61	0.24 (−0.69, 1.19)

*NRS nausea score*			
0–1 h	1.75 ± 2.88	1.48 ± 2.56	0.26 (−1.21, 1.74)
1–2 h	0.46 ± 1.03	1.07 ± 2.03	−0.60 (−1.47, 0.25)
2–4 h	0.25 ± 0.96	0.48 ± 1.36	−0.23 (−0.87, 0.40)
4–8 h	0.50 ± 1.62	0.85 ± 2.26	−0.35 (−1.41, 0.71)
8–12 h	0.42 ± 1.34	1.40 ± 3.01	−0.97 (−2.23, 0.27)
12–24 h	0.32 ± 1.36	0.40 ± 1.24	−0.08 (−0.79, 0.62)

Group O: ondansetron group; Group S: saline group; NRS: numeric rating scale; SD: standard deviation; CI: confidence interval.

## Data Availability

The data used to support the findings of this study are included within the article.

## References

[1] Stevens A. J., Woodman R. J., Owen H. (2015). The effect of ondansetron on the efficacy of postoperative tramadol: a systematic review and meta-analysis of a drug interaction. *Anaesthesia*.

[2] Beakley B. D., Kaye A. M., Kaye A. D. (2015). Tramadol, pharmacology, side effects, and Serotonin syndrome: a review. *Pain Physician*.

[3] Grond S., Sablotzki A. (2004). Clinical pharmacology of tramadol. *Clinical Pharmacokinetics*.

[4] Pang W. W., Mok M. S., Huang S., Hung S., Hung C. P., Huang M. H. (2000). Intraoperative loading attenuates nausea and vomiting of tramadol patient-controlled analgesia. *Canadian Journal of Anaesthesia*.

[5] Miotto K., Cho A. K., Khalil M. A., Blanco K., Sasaki J. D., Rawson R. (2017). Trends in tramadol. *Anesthesia & Analgesia*.

[6] Kovac A. L. (2018). Updates in the management of postoperative nausea and vomiting. *Advances in Anesthesia*.

[7] Kovac A. L. (2006). Prophylaxis of postoperative nausea and vomiting: controversies in the use of serotonin 5-hydroxytryptamine subtype 3 receptor antagonists. *Journal of Clinical Anesthesia*.

[8] Gan T. J., Diemunsch P., Habib A. S. (2014). Consensus guidelines for the management of postoperative nausea and vomiting. *Anesthesia & Analgesia*.

[9] Arcioni R., della Rocca M., Romanò S., Romano R., Pietropaoli P., Gasparetto A. (2002). Ondansetron inhibits the analgesic effects of tramadol: a possible 5-HT3 spinal receptor involvement in acute pain in humans. *Anesthesia & Analgesia*.

[10] De Witte J. L., Schoenmaekers B., Sessler D. I., Deloof T. (2001). The analgesic efficacy of tramadol is impaired by concurrent administration of ondansetron. *Anesthesia & Analgesia*.

[11] Cubukcu Z., Ozbek H., Gunes Y., Gunduz M., Ozcengiz D., Isik G. (2007). Effect of ondansetron in lower extremity bone surgery on morphine and tramadol consumption using patient controlled analgesia. *Agriculture*.

[12] Dürsteler C., Mases A., Fernandez V., Pol O., Puig M. M. (2006). Interaction between tramadol and two anti-emetics on nociception and gastrointestinal transit in mice. *European Journal of Pain*.

[13] Rauers N. I., Stüber F., Lee E.-H. (2010). Antagonistic effects of ondansetron and tramadol? a randomized placebo and active drug controlled study. *The Journal of Pain*.

[14] Prakash Yarramalle S., Munta K., Manimala Rao S., Venkategowda P. M., Sunka S., Kiran Dudam S. (2018). Comparision of analgesic efficacy of tramadol infusion versus tramadol plus ondansetron infusion in medical intensive care unit Indian. *Journal of Critical Care Medicine*.

[15] Raffa R. B. (2008). Basic pharmacology relevant to drug abuse assessment: tramadol as example. *Journal of Clinical Pharmacy and Therapeutics*.

[16] Vale C., Oliveira F., Assunção J., Fontes-Ribeiro C., Pereira F. (2011). Co-administration of ondansetron decreases the analgesic efficacy of tramadol in humans. *Pharmacology*.

[17] Erhan E., Onal A., Kocabas S., Parlar A., Yegul I., Kosay S. (2005). Ondansetron does not block tramadol-induced analgesia in mice. *Methods and Findings in Experimental and Clinical Pharmacology*.

[18] Yanarates O., Dogrul A., Yildirim V. (2010). Spinal 5-HT7 receptors play an important role in the antinociceptive and antihyperalgesic effects of tramadol and its metabolite, O-Desmethyltramadol, via activation of descending serotonergic pathways. *Anesthesiology*.

[19] Gibbison B., Bailey C. R., Klein A. A. (2015). Tramadol—the marmitedrug. *Anaesthesia*.

[20] Enggaard T. P., Poulsen L., Arendt-Nielsen L., Brøsen K., Ossig J., Sindrup S. H. (2006). The analgesic effect of tramadol after intravenous injection in healthy volunteers in relation to CYP2D6. *Anesthesia & Analgesia*.

[21] Stamer U. M., Musshoff F., Kobilay M., Madea B., Hoeft A., Stuber F. (2007). Concentrations of tramadol and O-desmethyltramadol enantiomers in different CYP2D6 genotypes. *Clinical Pharmacology & Therapeutics*.

[22] Stamer U. M., Stüber F. (2001). Analgesic efficacy of tramadol if coadministered with ondansetron. *Anesthesia & Analgesia*.

[23] Günaldı M., Erkisi M., Afşar C. U. (2014). Evaluation of endometrial thickness and bone mineral density based on CYP2D6 polymorphisms in Turkish breast cancer patients receiving tamoxifen treatment. *Pharmacology*.

[24] Golembiewski J. A., O’Brien D. J. (2002). A systematic approach to the management of postoperative nausea and vomiting. *Journal of PeriAnesthesia Nursing*.

[25] Apfell C. C., Roewer N. (2003). Risk assessment of postoperative nausea and vomiting. *International Anesthesiology Clinics*.

[26] Alghanem S. M., Massad I. M., Rashed E. M., Abu-Ali H. M., Daradkeh S. S. (2010). Optimization of anesthesia antiemetic measures versus combination therapy using dexamethasone or ondansetron for the prevention of postoperative nausea and vomiting. *Surgical Endoscopy*.

[27] Gan T. J., Meyer T., Apfel C. C. (2003). Consensus guidelines for managing postoperative nausea and vomiting. *Anesthesia & Analgesia*.

[28] (2004). *Zofran (Ondansetron Hydrochloride) Injection [package Insert]*.

